# Marginal Adaptation of Epoxy Resin and Calcium Silicate Sealers Under Two Obturation Techniques: A Scanning Electron Microscope Study

**DOI:** 10.7759/cureus.116783

**Published:** 2026-09-23

**Authors:** Sanmitra Saha, Somnath Pal, Binoy K Singh, Chi Koy Wang, Rishika Raman

**Affiliations:** 1 Department of Conservative Dentistry and Endodontics, Buddha Institute of Dental Sciences and Hospital, Patna, IND

**Keywords:** ah plus, ceraseal, continuous wave compaction, endodontics, marginal adaptation, root canal sealer, scanning electron microscopy, single-cone obturation

## Abstract

Background

A fluid-tight seal at the sealer-dentin interface prevents bacterial microleakage in endodontics. This in vitro study evaluated the marginal adaptation of an epoxy resin-based sealer (AH Plus, Dentsply DeTrey GmbH, Konstanz, Germany) and a calcium silicate-based sealer (CeraSeal, Meta Biomed Co., Ltd., Cheongju, South Korea) using continuous wave compaction (CWC) and single-cone (SC) obturation across root canal thirds using scanning electron microscopy (SEM).

Materials and methods

Sixty extracted human maxillary central incisors were standardized to 17 mm, instrumented with ProTaper Gold (Dentsply Maillefer, Ballaigues, Switzerland) up to F3, and allocated into four groups (n = 15 each): Group I (AH Plus + CWC), Group II (AH Plus + SC), Group III (CeraSeal + CWC), and Group IV (CeraSeal + SC). After incubation at 37°C in 100% relative humidity for seven days, specimens were sectioned at 3, 6, and 9 mm from the apex. Maximum interfacial gap widths (µm) were measured under SEM (500× magnification) by a calibrated examiner blinded to group allocations. Data were analyzed using two-way analysis of variance (ANOVA) and Tukey's post hoc tests (α = 0.05).

Results

Interfacial gap widths differed significantly across groups and canal thirds (p < 0.001). In the apical third, Group I showed the lowest gap (0.86 ± 0.42 µm), followed by Group II (2.04 ± 0.54 µm), Group III (3.87 ± 2.33 µm), and Group IV (5.70 ± 2.43 µm). In the middle third, Groups I and III exhibited superior adaptation. In the coronal third, Group III showed the lowest gap (1.51 ± 0.21 µm), whereas Group I had the highest gap separation (7.72 ± 3.80 µm).

Conclusions

AH Plus with CWC achieved superior adaptation in the apical and middle thirds, whereas CeraSeal with CWC performed best coronally. Overall, CWC produced more uniform marginal adaptation than the SC technique across both sealers.

## Introduction

Apical periodontitis is primarily caused by polymicrobial biofilms organized within an extracellular polymeric substance (EPS) matrix, which enables bacterial persistence within complex radicular niches [1]. Following chemo-mechanical disinfection and sonic activation to disrupt these biofilms, three-dimensional obturation is required to entomb residual microorganisms and seal the root canal terminus against fluid percolation and bacterial microleakage [2-5]. Because root canal sealers maintain prolonged contact with residual intratubular bacteria and may inadvertently contact periapical tissues, their biological profile and physicochemical stability are key considerations during material selection [5,6]. Modern calcium silicate-based bioceramic sealers exhibit favorable biocompatibility, promote osteogenic and cementogenic differentiation, and elicit minimal host inflammatory tissue reactions compared to traditional materials [7,8].

However, calcium silicate-based root canal sealers consistently exhibit higher solubility than conventional epoxy resin-based sealers such as AH Plus (Dentsply DeTrey GmbH, Konstanz, Germany), frequently exceeding the 3% mass loss limit set by ISO 6876:2012 standards; consequently, if extruded beyond the apex, material dissolution presents a distinct clinical consideration alongside osteogenic and cementogenic potential [9]. A comprehensive meta-analysis evaluating 22 laboratory investigations demonstrated that epoxy resin formulations exhibit significantly lower mass loss than calcium silicate-based alternatives, which frequently fail to meet the ISO threshold of less than 3% solubility [9]. This elevated solubility is inherent to the material class, as premixed calcium silicate sealers rely on the hydration of calcium silicate particles that produces calcium hydroxide, driving both alkaline ion release and dissolution [9].

Beyond biological attributes, the long-term clinical outcome of endodontic therapy is dictated by the physical integrity of the interfacial seal formed between the filling core, the sealer, and radicular dentin across different canal levels [5,10]. Deficiencies in this boundary manifest as micrometric interfacial gaps, forming pathways for microbial colonization and endodontic failure [11,12]. Historically, comparative evaluations have established that epoxy resin-based sealers, such as AH Plus, provide low solubility, favorable dimensional stability, and micromechanical adhesion through covalent bonding between active epoxide rings and exposed amino groups of dentinal collagen [10,13,14]. In contrast, premixed calcium silicate-based sealers (e.g., CeraSeal (Meta Biomed Co., Ltd., Cheongju, South Korea)) achieve chemical adhesion through the precipitation of an apatite-like crystalline layer, creating a distinct mineral infiltration zone along the tubular dentin perimeter [8,15].

Nevertheless, the final interfacial adaptation is fundamentally governed by the obturation technique employed [16,17]. Continuous wave compaction (CWC) utilizes thermal softening and vertical compaction forces to optimize hydraulic flow, compress sealer films, and obliterate micro-irregularities [17,18]. While CWC reliably enhances the adaptation of resin-based sealers, the thermal down-pack application poses potential challenges to calcium silicate sealers by accelerating hydration kinetics, altering moisture balance, and influencing interfacial stability [18,19]. Conversely, the hydraulic single-cone (SC) technique is widely advocated for bioceramics to circumvent heat-induced degradation; however, passive cone seating lacks active vertical hydraulic compaction, which may lead to excessive sealer film thickness, irregular void patterns, and compromised marginal adaptation in non-circular canal spaces [16,20].

High-resolution scanning electron microscopy (SEM) serves as a validated modality for micrometric visualization and direct quantification of interfacial gaps along radicular dentin at distinct anatomical levels [21,22]. Although the performance of epoxy resin and bioceramic sealers has been investigated under separate modalities, comparative cross-examinations evaluating the structural impact of thermal compaction versus cold hydraulic placement across coronal, middle, and apical horizons remain critical [17,23]. A distinct knowledge gap persists regarding whether the thermal and hydraulic forces of CWC compromise or enhance the interfacial adaptation of premixed calcium silicate sealers relative to cold SC placement across discrete radicular levels [17,18,20]. Therefore, this in vitro study utilized a standardized 2 × 2 factorial design to evaluate and compare the marginal adaptation of an epoxy resin-based sealer (AH Plus) and a calcium silicate-based bioceramic sealer (CeraSeal) to root canal dentin following CWC and SC obturation across the apical, middle, and coronal thirds using SEM. The null hypotheses tested were that (1) neither the sealer type nor the obturation technique would significantly influence interfacial marginal gap width and (2) no significant differences in marginal adaptation would occur across the different root canal thirds.

## Materials and methods

Study design

This in vitro experimental study was conducted between January 2026 and April 2026 at the Department of Conservative Dentistry and Endodontics of Buddha Institute of Dental Sciences and Hospital in Patna, Bihar, India. Ethical approval for the study protocol was obtained from the Institutional Ethics Committee of Buddha Institute of Dental Sciences and Hospital (approval number: 79/BIDSH/IEC/EXEMP/2024(2023-24)). SEM evaluation of the specimens was carried out at the Indian Institute of Technology (IIT), Bihta, Patna. Sample size estimation was performed prior to experimental procedures using the G*Power software (version 3.1.9.7; Heinrich-Heine-Universität Düsseldorf, Düsseldorf, Germany) based on effect sizes reported in previous SEM investigations assessing sealer interfacial adaptation [24]. Specifying an effect size (f) of 0.45, an alpha significance level of 0.05, and a statistical power (1 − β) of 0.80 across four independent experimental groups indicated a minimum requirement of 14 specimens per group [24]. To compensate for potential specimen attrition from microcracks during transverse sectioning, 15 specimens were allocated per group (total N = 60). Human maxillary central incisors were specifically selected to minimize morphological variability; their relatively straight, single radicular canal path (<5° curvature) provides a standardized anatomical model for assessing interfacial boundaries across root canal thirds [25].

Sample selection

Sixty freshly extracted human permanent maxillary central incisors with fully developed apices and a single straight root canal (<5° curvature) were collected from donors aged 20-45 years undergoing extractions indicated for severe periodontal compromise or orthodontic necessity at the Department of Oral and Maxillofacial Surgery. Written informed consent was obtained from all patients prior to tooth collection in accordance with institutional ethical oversight. All specimens were harvested within two months prior to experimental procedures, disinfected in 0.5% sodium hypochlorite solution for 30 minutes, rinsed thoroughly with distilled water, and stored in sterile physiologic saline at 4°C to prevent desiccation. Selected teeth were examined clinically and radiographically in buccolingual and mesiodistal projections to confirm a single canal without calcifications, internal or external resorption, craze lines, fractures, or previous endodontic intervention. Apical patency was verified by inserting a size #10 K-file (Mani Inc., Tochigi, Japan) through the apical foramen. Initial apical diameter was gauged using a size #15 K-file; specimens were included when a size #15 K-file traversed to the apical terminus whereas a size #20 K-file did not pass, establishing an initial apical diameter of less than 0.20 mm prior to mechanical shaping. Specimens exhibiting open apices, root curvatures exceeding 5°, or initial apical dimensions accommodating a size #20 file or larger were excluded. Adherent soft tissue remnants and calculus were removed using an ultrasonic scaler.

Sample preparation

Specimens were decoronated at the cemento-enamel junction using a low-speed diamond saw under continuous water cooling to obtain a standardized root length of 17 mm. Working length (WL) was established by inserting a size #10 K-file until visible at the major apical foramen and subtracting 1.0 mm. Canals were instrumented using the ProTaper Gold rotary nickel-titanium system (Dentsply Maillefer, Ballaigues, Switzerland) in an endodontic motor (X-Smart Plus; Dentsply Maillefer) operated at 300 rpm and 2.0 N·cm torque up to finishing file F3 (tip size 30, 0.09 taper at D1-D3) according to manufacturer recommendations. Preparing all canals uniformly to an F3 file established a standardized apical matrix matching the tip diameter and taper of corresponding gutta-percha master cones, ensuring reproducible hydraulic resistance across all groups. Canals were irrigated between successive instruments with 2 mL of 3% sodium hypochlorite using a 30-gauge side-vented needle placed 2 mm short of WL. Smear layer removal was achieved by flushing with 3 mL of 17% ethylenediaminetetraacetic acid (EDTA) for one minute, followed by acoustic activation with the SuperEndo Sonic Irrigation Activator (B&L Biotech, Tustin, CA, USA) equipped with a size #20, 0.02 taper tip operated at 10,000 cycles per minute for 30 seconds. Canals were flushed with 5 mL of sterile saline and dried with ProTaper Gold F3 absorbent paper points.

Group allocation

The 60 prepared specimens were randomized into four experimental groups (n = 15 each) through simple randomization using computer-generated random numbers (www.randomizer.org) according to the sealer and obturation technique employed: Group I (AH Plus sealer + CWC (AH-CWC)), Group II (AH Plus sealer + SC technique (AH-SC)), Group III (CeraSeal sealer + CWC (CS-CWC)), and Group IV (CeraSeal sealer + SC technique (CS-SC)).

Obturation procedure

In all experimental groups, a matched ProTaper Gold F3 gutta-percha master cone (Dentsply Maillefer) was seated to WL and verified for tactile tug-back. If tug-back was absent at WL, the apical tip of the cone was trimmed in 0.5 mm increments with a scalpel or reselected until definitive apical frictional resistance was confirmed.

In Groups I and III, obturation was performed via CWC. The respective sealer, AH Plus or CeraSeal, was introduced into the canal using the master cone to coat the walls. Continuous wave down-pack was carried out using the Fast-Pack Pro cordless thermal heating unit (Eighteeth, Changzhou Sifary Medical Technology Co., Ltd., Changzhou, China) fitted with an electric heat plugger (0.06 taper) set to 200°C, with the plugger advanced to 3-5 mm short of WL to compact the apical gutta-percha plug. The remaining canal space was backfilled using the Fi-G cordless gutta-percha delivery gun (Guilin Woodpecker Medical Instrument Co., Ltd., Guilin, China) delivering thermoplasticized gutta-percha at 200°C in sequential increments, with each increment compacted vertically using Buchanan hand pluggers.

In Groups II and IV, obturation was performed via the SC technique. The designated sealer was applied along the canal walls using the matched F3 master point, which was then seated passively to full WL without additional vertical compaction. The excess coronal cone was sheared off at the canal orifice using a heated instrument, and the surface was lightly adapted at the orifice entrance with a hand plugger.

Following obturation, all specimens were incubated at 37°C under 100% relative humidity for seven days to permit baseline setting of the sealers in accordance with ISO 6876:2012 protocols [2,20]. This incubation provides baseline dimensional stabilization necessary for sectioning and interfacial evaluation without risking dislodgement during specimen processing [20].

Sectioning of specimens

Following the incubation period, each root specimen was secured in a precision chuck and sectioned horizontally perpendicular to its long axis using a low-speed precision diamond saw (IsoMet; Buehler, Lake Bluff, IL, USA) under continuous sterile water irrigation. Three transverse sections (1.0 mm thickness) were obtained per specimen at 3 mm, 6 mm, and 9 mm from the root apex, representing the apical, middle, and coronal thirds, respectively. Slices were inspected under a stereomicroscope at 20× magnification; any slice displaying sectioning-induced microcracks, dentin fractures, or filling dislodgement was excluded and replaced with an intact reserve sample to ensure that 15 defect-free specimens were evaluated per group.

SEM evaluation

Transverse root slices were ultrasonically cleaned in distilled water for two minutes, dehydrated through an ascending ethanol series (50%, 70%, 90%, and 100% for 10 minutes each), air-dried, mounted on labeled aluminum stubs, and sputter-coated with a 10 nm gold layer using an automated sputter coater. Specimens were assigned randomized alphanumeric codes by an independent investigator to ensure examiner blinding. Imaging was performed using a ZEISS GeminiSEM 500 field emission SEM (Carl Zeiss, Oberkochen, Germany) operating at an accelerating voltage of 10-15 kV under 500× magnification. The sealer-dentin interface was evaluated across four circumferential quadrants for each section, and the maximum interfacial marginal gap width (µm) was measured using calibrated internal measurement software by a single calibrated examiner blinded to group allocations.

Statistical analysis

The recorded data were entered into Microsoft Excel (Microsoft Corp., Redmond, WA, USA) and analyzed using IBM SPSS Statistics for Windows, V. 26.0 (IBM Corp., Armonk, NY, USA). Data distribution was confirmed to be normal using the Shapiro-Wilk test, and homogeneity of variances was verified using Levene's test. Descriptive statistics were expressed as mean ± standard deviation (SD). Intergroup and inter-level comparisons were analyzed using two-way analysis of variance (ANOVA) to determine the main effects of sealer type, obturation technique, and their interactions across root thirds, followed by Tukey's post hoc honest significant difference (HSD) test for multiple pairwise comparisons. Statistical significance was set at α = 0.05.

## Results

Sixty extracted human permanent maxillary central incisors were randomly allocated into four experimental groups (n = 15) according to the sealer used and obturation technique employed: Group I (AH-CWC), Group II (AH-SC), Group III (CS-CWC), and Group IV (CS-SC). Following standardized obturation and storage under controlled conditions, specimens were sectioned at the apical (3 mm), middle (6 mm), and coronal (9 mm) levels and examined under SEM at 500× magnification to measure maximum marginal gap widths at the dentin-sealer interface. None of the experimental groups demonstrated a completely gap-free interface along the entire canal perimeter; both adapted segments and gap-containing regions were observed across all specimens. Intergroup and inter-level comparisons using two-way ANOVA revealed statistically significant differences in interfacial gap widths across groups and root canal thirds (p < 0.001).

At the apical third (3 mm), Group I (AH-CWC) demonstrated the lowest mean marginal gap value (0.86 ± 0.42 µm), followed by Group II (AH-SC; 2.04 ± 0.54 µm) and Group III (CS-CWC; 3.87 ± 2.33 µm), whereas Group IV (CS-SC) exhibited the highest mean marginal gap value (5.70 ± 2.43 µm) (Table 1).

**Table 1:** Mean marginal gap values (µm) at different root levels evaluated under SEM at 500× magnification. CWC: continuous wave compaction; SC: single-cone; SEM: scanning electron microscopy; SD: standard deviation; AH Plus: epoxy resin-based root canal sealer; CeraSeal: calcium silicate-based bioceramic root canal sealer

Group	Apical third (3 mm), mean	Apical third (3 mm), SD	Middle third (6 mm), mean	Middle third (6 mm), SD	Coronal third (9 mm), mean	Coronal third (9 mm), SD
Group I (AH Plus + CWC)	0.86	0.42	1.66	1.16	7.72	3.80
Group II (AH Plus + SC)	2.04	0.54	7.74	4.90	2.63	0.78
Group III (CeraSeal + CWC)	3.87	2.33	1.99	0.80	1.51	0.21
Group IV (CeraSeal + SC)	5.70	2.43	4.04	1.27	3.81	1.00

At the middle third (6 mm), Group I (AH-CWC) demonstrated the lowest mean marginal gap value (1.66 ± 1.16 µm), followed by Group III (CS-CWC; 1.99 ± 0.80 µm​​​​​​​) and Group IV (CS-SC; 4.04 ± 1.27 µm), while Group II (AH-SC) yielded the highest mean marginal gap value (7.74 ± 4.90 µm​​​​​​​) (Table 1).

At the coronal third (9 mm), Group III (CS-CWC) exhibited the lowest mean marginal gap value (1.51 ± 0.21 µm​​​​​​​), followed by Group II (AH-SC; 2.63 ± 0.78 µm) and Group IV (CS-SC; 3.81 ± 1.00 µm), whereas Group I (AH-CWC) demonstrated the highest mean marginal gap value (7.72 ± 3.80 µm​​​​​​​) (Table 1).

The overall distribution of marginal adaptation varied according to both sealer type and obturation technique (Table 2). Group I showed superior adaptation in the apical and middle thirds, whereas Group III showed superior adaptation in the coronal third. No single experimental group exhibited consistently superior marginal adaptation across all three evaluated canal horizons (Table 2).

**Table 2:** Overall trend of marginal adaptation according to sealer type and obturation technique. AH Plus: epoxy resin-based root canal sealer; CeraSeal: calcium silicate-based bioceramic root canal sealer

Group	Sealer	Obturation technique	Intragroup region of optimal adaptation
Group I	AH Plus	Continuous wave compaction	Apical and middle thirds
Group II	AH Plus	Single-cone	Coronal third
Group III	CeraSeal	Continuous wave compaction	Coronal third
Group IV	CeraSeal	Single-cone	Largest gap values across evaluated canal levels

Figures 1-4 depict the representative SEM micrographs of Groups I-IV evaluated across root canal thirds at 500× magnification.

**Figure 1:**
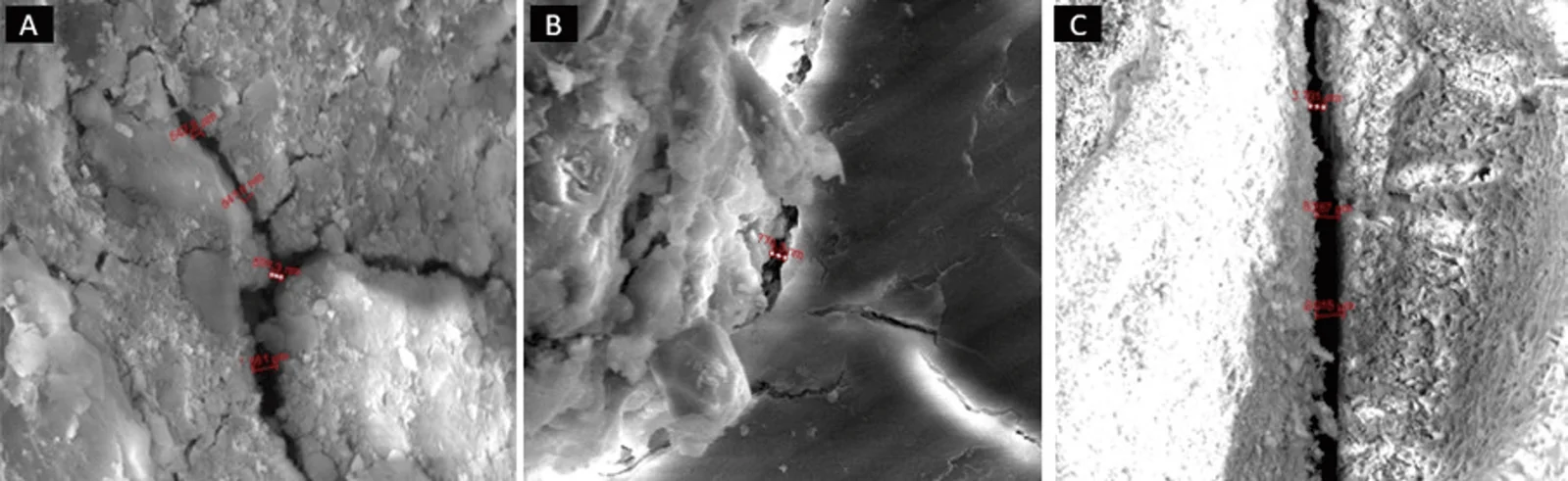
Representative SEM micrographs of Group I (AH-CWC: AH Plus with continuous wave compaction) evaluated at (A) apical (3 mm), (B) middle (6 mm), and (C) coronal (9 mm) thirds at 500× magnification. SEM: scanning electron microscopy

**Figure 2:**
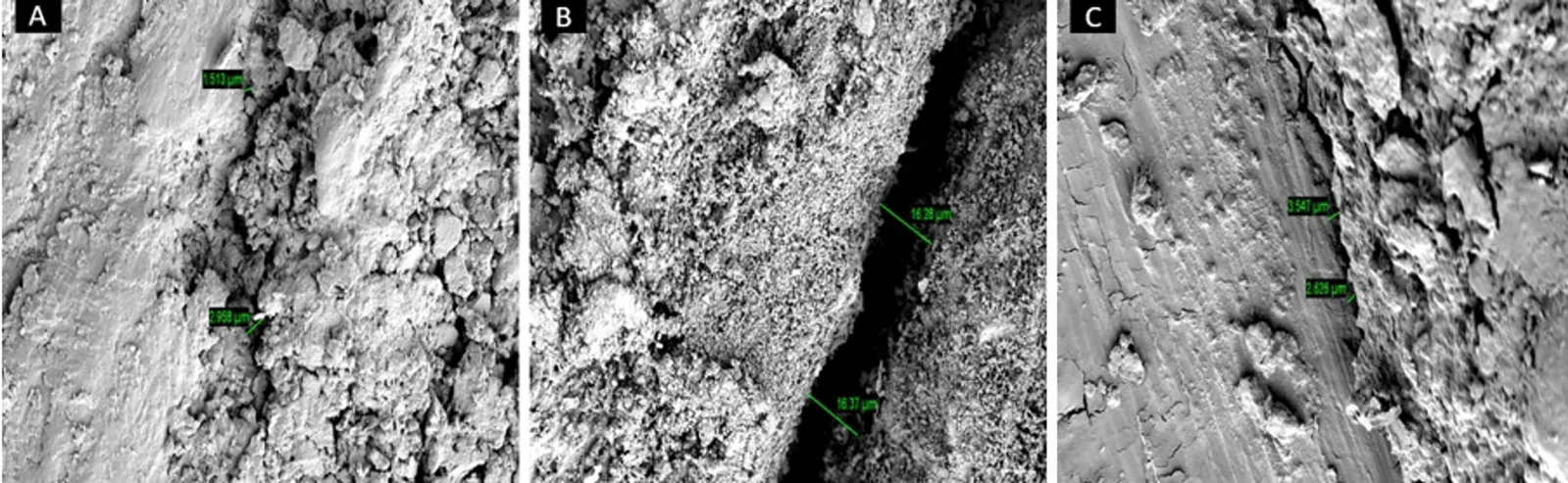
Representative SEM micrographs of Group II (AH-SC: AH Plus with single-cone technique) evaluated at (A) apical (3 mm), (B) middle (6 mm), and (C) coronal (9 mm) thirds at 500× magnification. SEM: scanning electron microscopy

**Figure 3:**
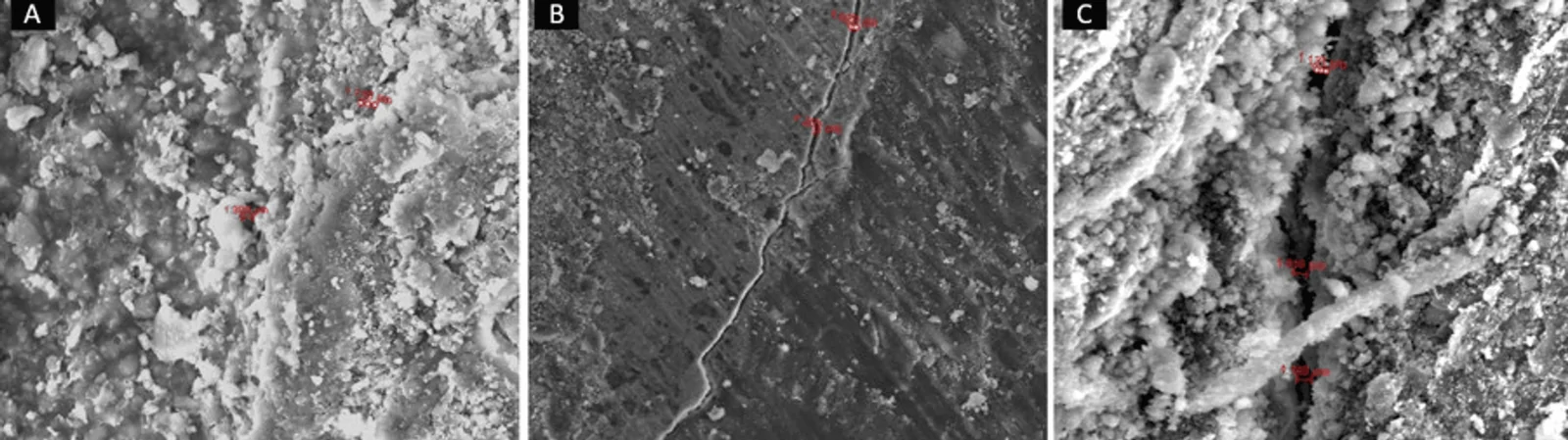
Representative SEM micrographs of Group III (CS-CWC: CeraSeal with continuous wave compaction) evaluated at (A) apical (3 mm), (B) middle (6 mm), and (C) coronal (9 mm) thirds at 500× magnification. SEM: scanning electron microscopy

**Figure 4:**
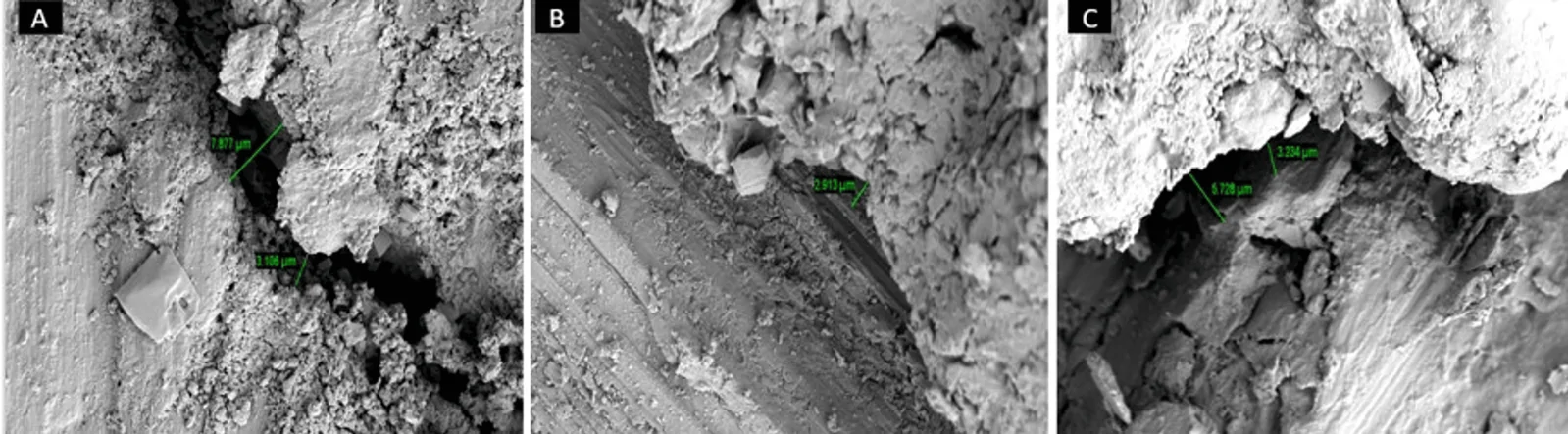
Representative SEM micrographs of Group IV (CS-SC: CeraSeal with single-cone technique) evaluated at (A) apical (3 mm), (B) middle (6 mm), and (C) coronal (9 mm) thirds at 500× magnification. SEM: scanning electron microscopy

## Discussion

The primary objective of non-surgical endodontic therapy is the maximal reduction of intra-radicular microbial populations to a threshold compatible with periapical tissue healing and the prevention of subsequent reinfection through three-dimensional obturation [1,2]. Achieving an adequate seal against radicular microleakage is necessary to minimize fluid percolation and entomb residual microorganisms surviving within complex anatomical niches [2,5]. The critical weak link in this sealing mechanism remains the interface between the root canal filling and radicular dentin, where the configuration, flow, and dimensional stability of the sealer determine the occurrence of interfacial gaps [10,11]. The present in vitro investigation comparatively evaluated the marginal adaptation of an epoxy resin-based sealer (AH Plus) and a calcium silicate-based bioceramic sealer (CeraSeal) utilizing CWC and SC techniques across standardized human maxillary central incisors. Based on SEM evaluation, both null hypotheses were rejected, as significant differences in marginal gap widths were observed across the tested sealers, obturation techniques, and root canal levels (p < 0.001).

The interfacial analysis revealed distinct micrometric variations among all experimental groups. A primary finding of this study is that AH Plus combined with CWC (Group I: AH-CWC) achieved the lowest mean marginal gap values in both the apical (0.86 ± 0.42 µm) and middle thirds (1.66 ± 1.16 µm), demonstrating superior interfacial adaptation. This observation aligns with the findings of Polineni et al. [14], who evaluated sealer adaptation under SEM and reported minimal interfacial discrepancies with epoxy resin formulations. AH Plus exhibits favorable flow, low solubility, and dimensional stability, which facilitate surface wetting under compaction forces [13,14]. Furthermore, the adhesion of AH Plus is reinforced by covalent bond formation between active epoxide rings and open amino groups in the collagenous dentin matrix [16]. Under the hydraulic forces of CWC down-pack, thermoplasticized gutta-percha drives the resin sealer into microscopic dentinal irregularities, establishing a thin, uniform film that minimizes polymerization shrinkage stresses in confined apical spaces [16,18]. This mechanical interlocking and cohesive stability explain its favorable sealing efficiency when compacted thermally [26,27].

In single-rooted maxillary central incisors, root canal morphology frequently transitions from a round configuration apically to an ovoid geometry in the middle segment. While rotary preparation up to an F3 finishing file establishes a standardized apical matrix, circular rotary instrumentation primarily prepares the central canal path, leaving uninstrumented lateral extensions within the ovoid middle third. Under CWC, thermoplasticized gutta-percha and compaction forces generate hydraulic vectors that drive the resin sealer into these irregular anatomical recesses. Conversely, the passive SC technique relies strictly on a standardized round master cone, which exerts no lateral compaction forces. Consequently, in Group II (AH-SC), excessive pools of uncompacted resin accumulated within these uninstrumented recesses in the middle third, manifesting the highest mean marginal gap at this level (7.74 ± 4.90 µm) as polymerization shrinkage stresses caused localized detachment from the dentinal walls [12,14,16].

In the coronal third, AH Plus combined with CWC (Group I; 7.72 ± 3.80 µm) exhibited the largest gap width among all coronal cohorts (p < 0.001). This localized detachment corroborates the findings of Jain and Adhikari [12] and Srikumar et al. [11], who observed that epoxy resin sealers exhibit wider gaps in broader canal dimensions where larger volumes of material reside. During continuous wave backfilling, thermoplasticized gutta-percha undergoes approximately 1-2% volumetric thermal shrinkage upon cooling within the wide coronal lumen [18,19]. In this wide segment, characterized by an unfavorable configuration factor (C-factor), thermal contraction of the core mass combined with polymerization shrinkage of thick sealer layers promotes interfacial detachment along the tubular dentin perimeter [11,12].

In contrast, the premixed calcium silicate bioceramic sealer paired with CWC (Group III: CS-CWC) achieved the lowest marginal gap value in the coronal third (1.51 ± 0.21 µm). This coronal behavior is supported by the setting characteristics of calcium silicate materials. Premixed bioceramic sealers such as CeraSeal utilize dentinal moisture to initiate their hydration cascade [15,17]. During hydration, calcium silicate reacts with water to form a calcium-silicate-hydrate gel while precipitating calcium hydroxide, which subsequently interacts with environmental phosphate ions to form an interfacial apatite crystalline layer along the tubular dentin perimeter [8,28]. This mineral infiltration zone provides dimensional stability and minimal setting shrinkage [15,20]. In the wide coronal third, moisture availability from patent, large-diameter dentinal tubules supports this crystalline hydration, producing a stable interfacial seal that resists detachment [17,20]. These interfacial interactions contribute directly to the variable tubular penetration patterns observed between resin and bioceramic formulations under hydraulic delivery [29,30].

However, when CeraSeal was utilized in the apical third via the SC technique without heat (Group IV: CS-SC), it generated the widest mean marginal gap value (5.70 ± 2.43 µm) across all apical cohorts. While previous investigations by Wael et al. [15] and Najafzadeh et al. [31] noted dentinal tubule penetration with SC bioceramic obturation, the present data confirm that intratubular tag penetration does not inherently assure continuous marginal gap-free adaptation along the canal perimeter [32]. In the constricted apical third, passive SC placement generates minimal hydraulic pressure, frequently leaving unadapted sealer reservoirs or voids along uninstrumented anatomical irregularities [16,20]. Furthermore, reduced tubule density and lower moisture availability in the apical third may prolong setting kinetics, increasing material vulnerability to gap formation prior to complete crystalline maturation [28,33].

The increased solubility of calcium silicate sealers also creates a physiological paradox: while dissolution permits bioactivity and mineral precipitation that supports the dentinal interface, it concurrently compromises the non-reactive gutta-percha interface, elevating the susceptibility to interfacial gap formation and long-term fluid microleakage [34-36]. Because gutta-percha is an inert polyisoprene core that forms no chemical adhesion with calcium silicates, material shrinkage or partial dissolution during the setting phase creates localized voids along the core boundary [34,35].

The introduction of CWC to CeraSeal (Group III: CS-CWC) improved apical adaptation (3.87 ± 2.33 µm) compared to its SC counterpart (5.70 ± 2.43 µm), supporting the principle that vertical compaction forces assist in spreading viscous materials into fine radicular discrepancies [16,37]. Nevertheless, apical gap widths in Group III remained significantly wider than those recorded for AH Plus under CWC (0.86 ± 0.42 µm). This finding highlights the thermal sensitivity of calcium silicates; high down-pack temperatures can cause rapid desiccation of internal water, alter setting kinetics, and induce microstructural changes near the heat-carrier boundary [18,19]. Such thermal alterations may also explain discrepancies in dislodgement resistance, push-out bond strength, and retreatability reported when bioceramic sealers are subjected to warm vertical compaction compared to cold techniques [38,39]. Although favorable clinical and radiographic outcomes have been documented for both resin-based and bioceramic sealers in practice [40], microscopic gap formation along radicular walls remains a principal pathway for fluid percolation and apical leakage [41].

While the seven-day incubation period employed in this study provided baseline stabilization in line with ISO 6876 standards, the setting reaction of calcium silicate sealers is recognized to be a continuous, prolonged process [20]. Premixed calcium silicate-based materials undergo progressive physicochemical maturation, ion release, and dynamic phase transformations over extended periods [42-44]. Consequently, the interfacial gap widths recorded at seven days represent an early post-obturation baseline; long-term aging under aqueous conditions may induce further interfacial precipitation and gap closure, an aspect that warrants longitudinal validation [43,44].

Limitations

Several limitations must be acknowledged when interpreting these findings. First, as an in vitro laboratory study, this model does not replicate dynamic clinical conditions, including the continuous outward hydraulic pressure of dentinal fluid, intra-pulpal temperature fluctuations, and cyclic masticatory loads. Second, this investigation utilized sound extracted teeth maintained in a neutral storage environment, whereas in clinical scenarios presenting with persistent apical periodontitis, sealers are frequently exposed to an acidic inflammatory microenvironment [45]. Lower environmental pH can significantly impair the hydration kinetics of calcium silicate materials, compromising their surface microhardness, elastic modulus, and structural density while increasing porosity [45]. Third, the study was conducted exclusively on straight, single-rooted maxillary central incisors to standardize canal anatomy; hence, findings cannot be directly extrapolated to complex root canal systems exhibiting severe curvatures, lateral anastomoses, or irregular, flattened canal configurations [25]. Fourth, the high-vacuum dehydration and gold sputter-coating protocols necessary for conventional SEM examination carry an inherent risk of inducing artifactual desiccation microcracks along brittle interfacial boundaries, which may accentuate measured gap dimensions [33]. Finally, while SEM provides high-resolution two-dimensional cross-sectional visualization of marginal boundaries at discrete root thirds, it is fundamentally a destructive technique that cannot assess continuous three-dimensional void volumes throughout the full canal space [17,21]. In this context, high-resolution micro-computed tomography (micro-CT) provides a validated, non-destructive volumetric methodology capable of quantitatively mapping total, open, and closed void distributions across the entire obturation mass over long-term evaluation intervals, serving as an essential complementary tool for comprehensive sealing assessments [46,47].

## Conclusions

Within the parameters and limitations of this in vitro investigation, both root canal sealer chemistry and obturation technique significantly influenced marginal adaptation at the sealer-dentin interface. AH Plus combined with CWC yielded the lowest interfacial gap values in the apical and middle thirds, demonstrating superior marginal adaptation in these segments. In contrast, CeraSeal combined with CWC exhibited the lowest interfacial gap values in the coronal third. Complete gap-free adaptation along the entire canal perimeter was not achieved in any experimental cohort, indicating that anatomical variations and compaction dynamics inherently govern interfacial sealing behavior. Rather than providing an absolute impervious seal, endodontic obturation systems remain constrained by material handling properties and radicular geometry across different canal horizons. Longitudinal assessments and non-destructive three-dimensional micro-CT investigations are warranted to evaluate volumetric void distributions and corroborate these interfacial observations with clinical outcomes.
